# Quantification of Microbial Robustness in Yeast

**DOI:** 10.1021/acssynbio.1c00615

**Published:** 2022-03-11

**Authors:** Cecilia Trivellin, Lisbeth Olsson, Peter Rugbjerg

**Affiliations:** † Department of Biology and Biological Engineering, Division of Industrial Biotechnology, Chalmers University of Technology, Gothenburg 412 96, Sweden; ‡ Enduro Genetics ApS, Copenhagen 2200, Denmark

**Keywords:** robustness quantification, phenomics, high-throughput, yeast, bioprocess, Fano factor

## Abstract

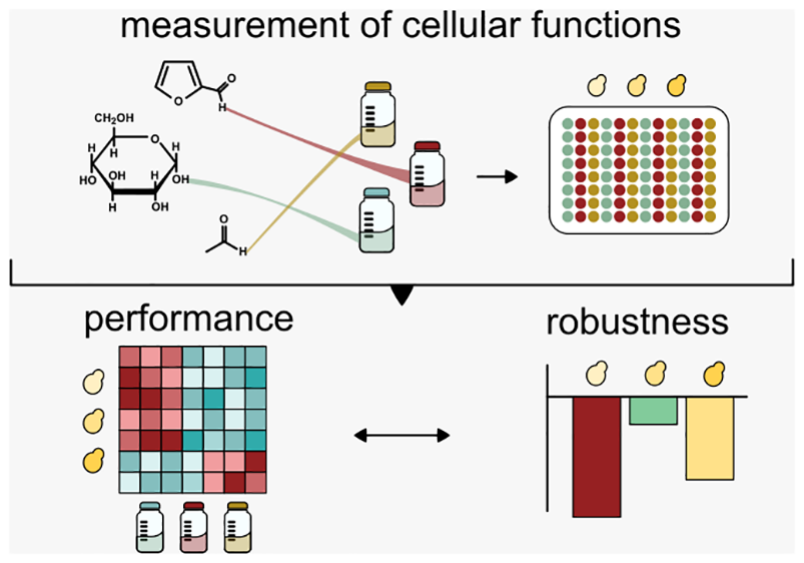

Stable cell performance in a fluctuating environment is essential for sustainable bioproduction and synthetic cell functionality; however, microbial robustness is rarely quantified. Here, we describe a high-throughput strategy for quantifying robustness of multiple cellular functions and strains in a perturbation space. We evaluated quantification theory on experimental data and concluded that the mean-normalized Fano factor allowed accurate, reliable, and standardized quantification. Our methodology applied to perturbations related to lignocellulosic bioethanol production showed that the industrial bioethanol producing strain *Saccharomyces cerevisiae* Ethanol Red exhibited both higher and more robust growth rates than the laboratory strain CEN.PK and industrial strain PE-2, while a more robust product yield traded off for lower mean levels. The methodology validated that robustness is function-specific and characterized by positive and negative function-specific trade-offs. Systematic quantification of robustness to end-use perturbations will be important to analyze and construct robust strains with more predictable functions.

Microbial robustness ensures predictable, stable synthetic cellular functionality in spite of internal or external perturbations.^1−3^ Robust cell manufacturing and destination performance will be a key in realizing new synthetic biology modalities and efficient bioproduction.^4−6^ Robustness is defined for a specific function (or phenotype) and set of perturbations.^7^ Robustness is therefore different from tolerance, which specifically describes stable growth or survival to various perturbations, e.g., via specific growth rates.^6,8^ In silico systems biology quantifies robustness by the influence on a cellular function of a frequency-normalized perturbation space relative to a control condition^9^ with a normally distributed mean and standard deviation. Experimentally, measures of variation represent the stability (dispersion) of quantitative traits across perturbations, but not at different scales,^7^ for which the dimensionless coefficient of variation (CV) is better suited.^10^ Yet even if central to realizing predictable, scalable synthetic biology, robustness is seldomly quantified experimentally for strain functions, which may be subject to genetic or environmental perturbations of stochastic or determined behavior.^5,11,12^ Here, we present and validate a high-throughput methodology to experimentally quantify microbial robustness (script available on GitHub). We show that the methodology can be used to systematically quantify and compare the robustness of different strain functions of interest in a relevant perturbation space and relate them to their performance level. Precise quantification will allow for exploration of trade-offs between robustness and performance of different cellular functions.

## Results and Discussion

### Development of a Systematic Method for Quantification of Robustness Based on the Fano Factor

In order to experimentally quantify robustness (*R*), we first set four important criteria to ensure consistency, reproducibility, and standardization. (1) Testing more perturbations should not change *R*, only its statistical significance. (2) Positive and negative deviations from the mean or a control performance level^9^ should contribute negatively to *R*. (3) Higher *R* should represent greater robustness. (4) *R* should be dimensionless and capture cellular function values at different orders of magnitude allowing comparison. To meet these criteria, we evaluated the reported theory.^9,10^

The first theory quantifies *R* as the CV based on standard deviation/mean (σ/*x̅*).^10^*R*_CV_ was calculated as
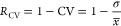
1However, when cellular functions are subjected to different experimental perturbations, CV becomes >1 complicating interpretation. More importantly, as others have, we found that CV was poorly accurate in describing data dispersion with means between 0 and 1 (Figure S1).^13^ CV therefore failed our fourth criterion.

The second theory quantifies *R* by evaluating a function change in relation to a specific control condition 0, according to an embodiment of Kitano’s formula:^9^
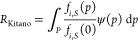
2

*R*_Kitano_ reports the ratio between the perturbed function *f*_*i*__,*S*_(*p*) and the control condition *f*_*i*__,*S*_(0) over a space of perturbations *P*, each multiplied for its frequency ψ(*p*). We simplified eq 2 and assumed an equal frequency for each perturbation. However, functions performing better than the control achieved higher robustness (Figure S2); further, defining a control condition performance is not always meaningful. As a result, *R*_Kitano_ failed our criteria 1, 2, and 4.

**Figure 1:**
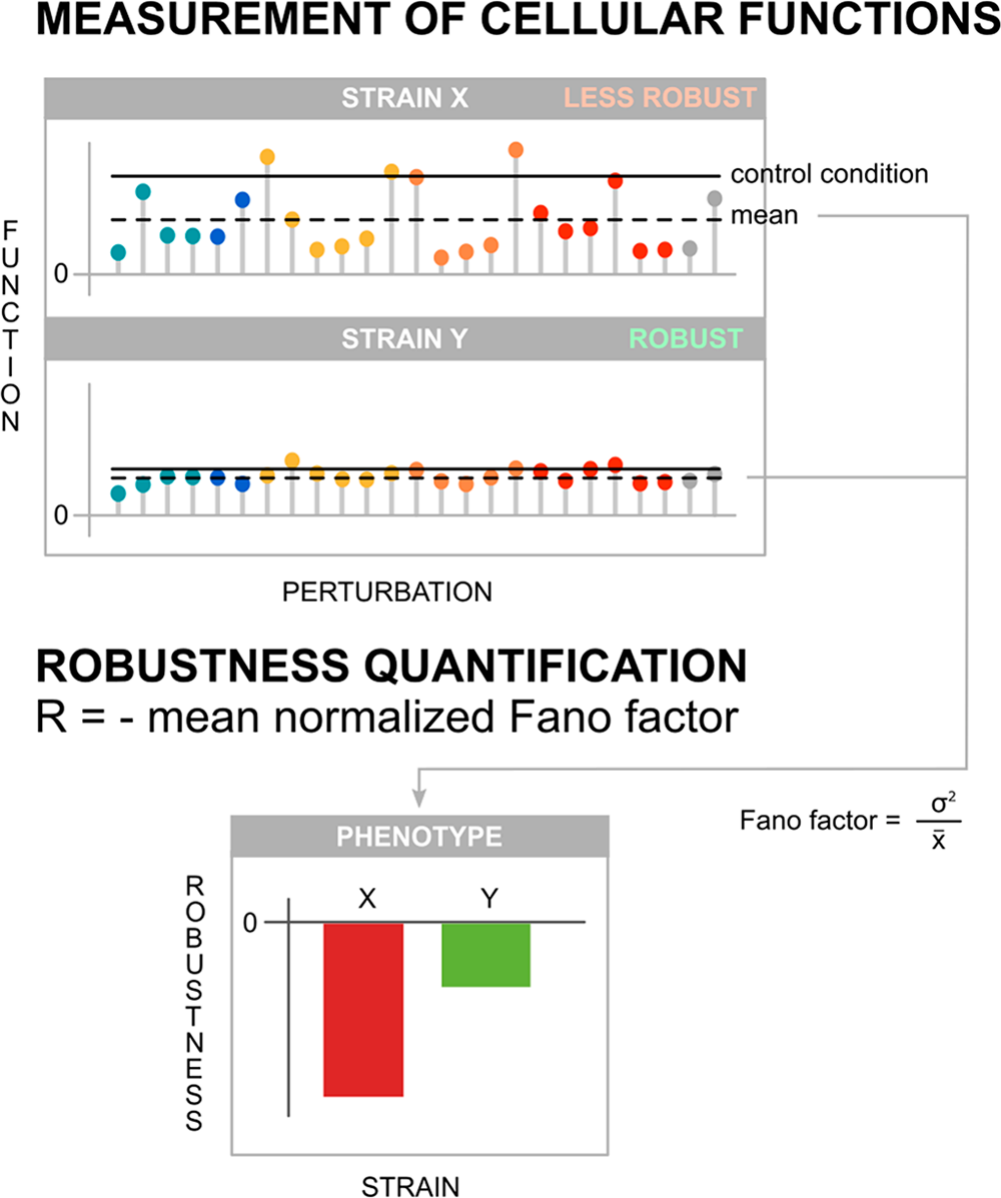
Relevant functions are measured upon exposure to various perturbations (colored dots) and robustness is calculated as the negative mean-normalized Fano factor. A control condition (e.g., 20 g/L glucose) is needed for calculation of *R*_Kitano_.

Therefore, we evaluated an approach quantifying *R* as the dispersion of data around the function means using the Fano factor (Figure 1). The Fano factor is commonly used to study transcriptional bursting and noise in gene expression by identifying deviation from Poissonian behavior and has been proposed for robustness before,^7,13−15^ but not actually deployed. For each function *i*, a strain *S*, and a perturbation space *P*, *R*_*S*,*i*,*P*_ was calculated as σ^2^/*x̅*:^16^
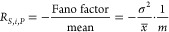
3To allow comparison of *R* between functions, we normalized the different Fano factors to the mean of the functions they describe (*m*) across all strains (Figure 1). We set the upper limit for *R* to 0 (highest robustness) and the problem of 0 < *x̅* < 1 was solved. As the Fano factor remained finite for mean values approaching zero, the weight of the mean on *R* was higher than for the CV. This quantification strategy was frequency independent, dimensionless, and free from arbitrary control conditions, thereby meeting all criteria. The use of *m*, however, still means that the *R* values calculated using the mean-normalized Fano factor by definition always will be relative to the investigated data case.

### Quantifying Robustness of Five Cellular Functions: Case Study of Lignocellulosic Bioethanol Production

To validate the methodology for quantifying microbial robustness, we used lignocellulosic bioethanol production (Materials and Methods). We included the *Saccharomyces cerevisiae* CEN.PK113–7D laboratory strain,^17^ and the industrial strains Ethanol Red and PE-2, whose robust ethanol production and growth is advantageous in starch and sugar cane fermentation.^18−20^ We measured five relevant cellular functions (maximum specific growth rate, lag phase, cell dry weight, biomass, and ethanol yields) across 29 different perturbations (single-component lignocellulose growth conditions) in a 96-well plate high-throughput setup (Materials and Methods).

**Figure 2:**
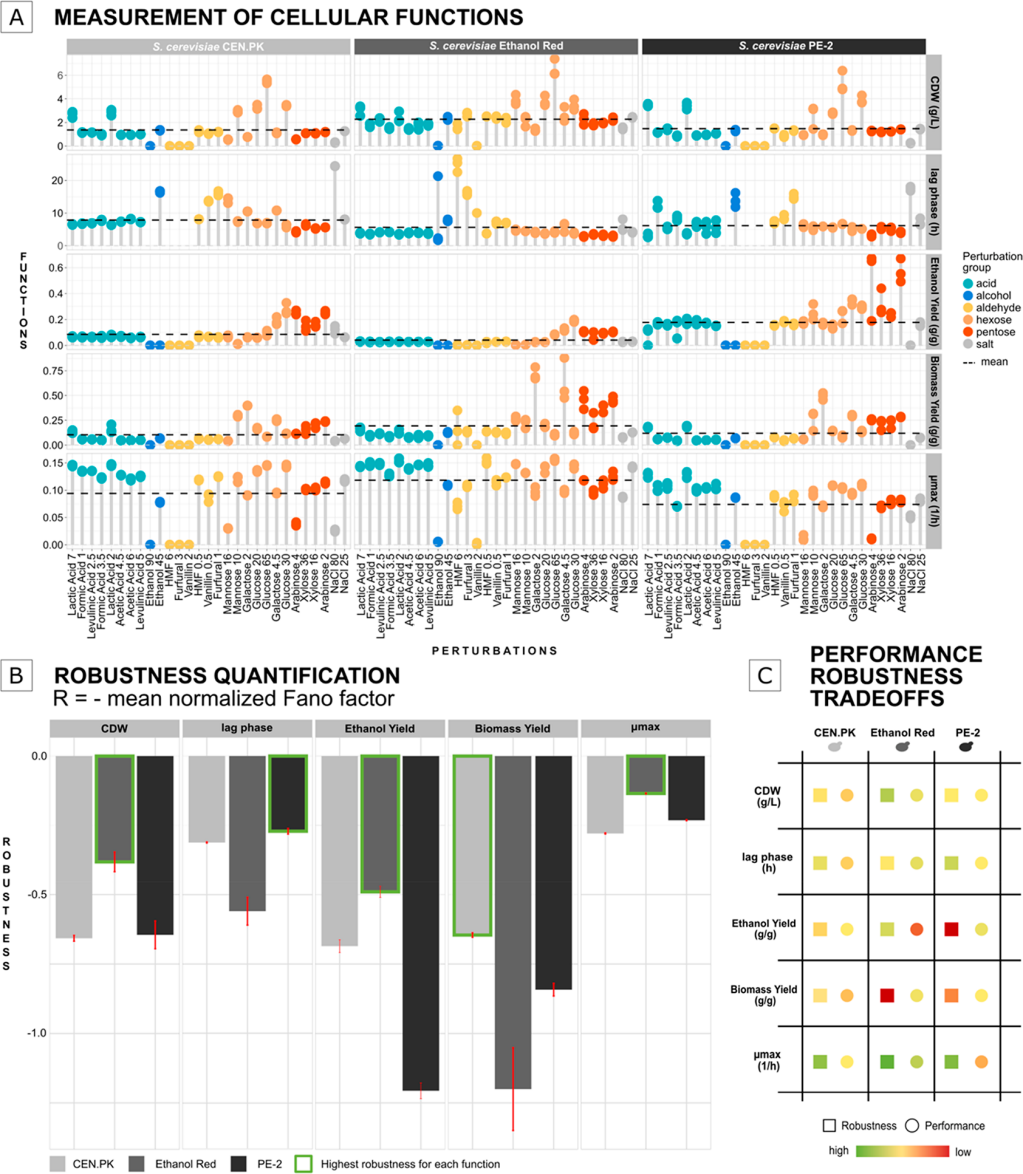
Quantification of microbial robustness with the Fano factor. (A) Function evaluation in a large perturbation space containing components found in lignocellulosic hydrolysates. CDW: cell dry weight; μ_max_: maximum specific growth rate. All points are individual biological replicates (*n* = 3). Lag phase missing points: cultures did not grow within 48 h. (B) Robustness quantification for each function. Error bars: standard error of the mean (*n* = 3). (C) Robustness and performance trade-offs for each function and strain.

The three strains exhibited different production and growth functionality when exposed to the 29 perturbations (Figure 2A). Across the lignocellulose perturbation space, Ethanol Red performed better than CEN.PK and PE-2 in all functions, except for ethanol yield. Aldehydes had a negative effect on all five measured functions, while pentoses resulted in unchanged or improved functions. Lactic, levulinic, formic, and acetic acid reduced cell dry weight and biomass yield (Figure 2A).

We next quantified the robustness and found the maximum specific growth rate, ethanol yield, and cell dry weight as significantly higher in Ethanol Red (cell dry weight (*p*-value < 0.005), maximum specific growth rate (*p*-value < 7 × 10^–8^), ethanol yield (*p*-value < 0.001)) (*t* test) (Figure 2B), supported by data less dispersed around the mean. These robust cellular functions were accompanied by a more fragile biomass yield, most robust in CEN.PK (*p*-value < 0.02), and lag phase most robust in PE-2 (*p*-value < 0.002) (Figure 2B). PE-2 achieved high mean ethanol yield, but its robustness was the lowest in part due to positive effects from pentoses. The observed very robust growth and production functions of Ethanol Red could explain its application in first-generation bioethanol plants that share our perturbations mainly except aldehydes,^18^ but came with a cost of lower average performance. High performance and high robustness are sometimes considered mutually exclusive.^21^ In theory, these two properties are believed to trade off with one another in several biological systems.^22,23^ Our quantification methodology identified possible robustness and performance trade-offs in lignocellulose-based bioethanol production (Figure 2C). For example, Ethanol Red traded its ethanol yield performance for high robustness, and vice versa for PE-2. Oppositely, we found that PE-2 traded robustness for performance for its maximum specific growth rate. On the basis of these findings, we observed that correlations between performance and robustness are both function and strain dependent. Larger data sets are needed to establish such correlations. In our investigation, the mean-normalized Fano factor was the best option to calculate robustness, but we noticed that its suitability could be limited by the normalization with *m* (the mean of the function performance among the strains). The normalization is required by criteria four; however, it further adjusts *R* to the strains tested in the study.

A systematic framework for assessing robustness of several performance indicators will improve our understanding of how cellular functions respond to relevant perturbations, e.g., by favoring robustness, performance, or a suboptimal state for both. In strain engineering, robust yields of products are sometimes preferred over higher but unstable yields.^5^ In synthetic biology, engineered strains should carry new functions that perform robustly under anticipated perturbations. Relevant functions include biosensor signals, gene expression reporters and heterologous proteins production. Quantifying robustness to scale-up or long-term cultivation (such as genetic robustness) will also be highly relevant to prevent declines of performance due to accumulation of mutations and heterogeneity,^5,24^ e.g., by quantifying robustness over many cellular divisions.

The high-throughput methodology described here will be useful for quickly quantifying robustness of multiple strain variants, environments and functions. For example, when engineering strains for heterologous expression, one could compare the robustness and performance trade-offs of product yield under various environmental and genetic conditions (e.g., different gene homologues, promoters, growth conditions). By doing so at laboratory scale, it may be possible to better understand and screen for robust cellular functions, rather than high-performing but unstable functions. When a strain is selected with a robust cell function, shifting conditions (e.g., different concentrations of inhibitors in the growth media) would not affect functionality. Thus, different industrially “robust strains” may be optimized toward robustness of production functionality across the narrow environmental and stochastic perturbation space of the bioprocess, making such strains robust to typical fluctuations in the process and thus not necessarily robust in foreign environments. Quantification methodology can uncover which perturbations affect the robustness of specific functions and how robustness relates positively and negatively to performance for each function (i.e., trade-offs). Through our methodology, we validated that robustness is function-specific,^7^ rather than a universal strain value, as previously theorized.

Future work will also show whether strains with robust functions are better starting points for subsequent enhancements via metabolic or evolutionary engineering. Robustness quantification may also include perturbation frequency, as well as more strains and perturbations. At present, our methodology may be applied to phenomics databases to compare numerous traits and strains.

## Material and Methods

### Strains

The strains used in the study were *Saccharomyces cerevisiae* CEN.PK113–7D^25^ (Scientific Research and Development GmbH, Oberursel, Germany), PE-2^26^ (wild-type strain isolated during sugar cane-to-ethanol production in Brazil), and Ethanol Red (kindly provided by Société Industrielle Lesaffre, Division Leaf).

### Media Preparation

Delft minimal medium^27^ was used for strain cultivation. The medium was prepared with 5 g/L (NH_4_)_2_SO_4_, 3 g/L KH_2_PO_4_, 1 g/L MgSO_4_·7H_2_O, 1 mL (in 1 L solution) trace mineral solution (Table S1), and 1 mL (in 1 L solution) vitamin solution (Table S2). The medium was adjusted to pH 5 with KOH and buffered with 250 mM potassium hydrogen phthalate. Multiple compounds were added to the minimal medium to mimic the composition of lignocellulosic hydrolysates obtained mostly from spruce, corn starch, and wheat straw (Table S3).

### Fermentation Experiments

The strains were preserved in glycerol (final concentration 16%) at −80 °C. Precultures were prepared by inoculating 10 μL glycerol stocks in 5 mL of the above-described Delft medium (20 g/L glucose), followed by incubation at 30 °C with 200 rpm shaking.

The optical density at 600 nm (OD_600_) of overnight cultures was monitored in a GENESYS 10 spectrophotometer (Thermo Scientific). After 24 h, the cultures (in exponential phase) were inoculated in square polystyrene 96-half-deepwell microtiter plates (CR1496dg; Enzyscreen) at a starting OD_600_ of 0.02. Strain growth was monitored in a Growth Profiler 960 (Enzyscreen) and was expressed as green value (GV) units. The microplates were covered with a CO_2_-release cover (CR1296t; Enzyscreen) to minimize passive diffusion of O_2_ and mimic anaerobic conditions. Each plate was used for a single strain, each well corresponded to a specific growth condition, and each condition was assayed in three technical replicates. The strains were cultivated in the growth profiler for 48 h at 30 °C, with 250 rpm shaking. After 48 h, the plates were removed from the growth profiler and the GV units at 48 h were converted to OD values according to the following formula:
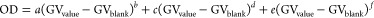
The constants were as follows: *a* = 0.019, *b* = 1, *c* = 3.82 × 10^–6^, *d* = 2.66, *e* = 3.111 × 10^–22^, *f* = 10.5, and GV_blank_ = 26.3.

The equation was previously calibrated using Delft medium and *S. cerevisiae* CEN.PK113–7D. Cultures were diluted based on the final OD_600_ measured in the growth profiler. OD_600_ of the culture at 48 h was measured in a plate reader (SPECTROstar nano; BMG LABTECH). The culture was transferred on a hydrophilic polytetrafluoroethylene multiscreen solvinert 96-well filter plate (MSRLN0410; Millipore) and filtered into a new 96-well microtiter plate (82.1581; Sarstedt).

### Cell Dry Weight (CDW) Determination

*S. cerevisiae* strains were cultivated in Delft medium (20 g/L glucose) to stationary phase and centrifuged at 5000 rpm for 5 min. The cell pellet was resuspended in 1 mL water and OD_600_ was measured. A series of five 1:2 dilutions were made and filtered through a predried and weighed 0.45 μm poly(ether sulfone) membrane (Sartorius). OD_600_ of the dilutions was measured. The filters containing the samples were dried for 10 min in a microwave oven (350 W) and weighed again. Calibration curves were constructed with CDW and OD_600_. The slope values for each strain were used subsequently to calculate the CDW of the growth profiler cultures.

### Determination of Sugars and Ethanol

Culture medium obtained by filtration from the first cultivation was used to determine the sugars and ethanol content. Ethanol was measured with the K-ETOH Ethanol Assay Kit, glucose with the K-GLUHK-220A d-Glucose HK Assay Kit, mannose with the K-MANGL d-Mannose/d-Fructose/d-Glucose Assay kit, xylose with the K-XYLOSE d-Xylose Assay Kit, and galactose and arabinose with the K-ARGA l-Arabinose/d-Galactose Assay Kit (all Megazyme). The assays are based on enzymatic reactions, which produce NADH, whose absorbance at 340 nm can be read in a spectrophotometer. The amount of NADH is stoichiometric with the amount of the compound of interest, making it possible to calculate the concentration (g/L) of the respective compounds.

### Determination of Performance Values

The growth data in GV units were imported in R software for visualization and determination of growth parameters. The maximum specific growth rate (μ_max_) was determined using all*_splines* function. Duration of the lag phase was determined by calculating the *x* coordinate of the intersection between the line with μ_max_ slope passing through the inflection point and the line passing through *y*0 parallel to the *x*-axis. In the wells where no growth was detected, *R*^2^ was <0.99, so the μ_max_ was set to 0 while the lag phase was set to NA.

Ethanol yield and biomass yield were calculated based on total consumed sugars as follows:
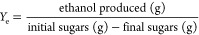
4
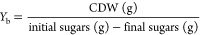
5Scripts with line-by-line explanation available on Github (https://github.com/cectri/Quantification-of-microbial-robustness).

### Robustness Calculation

Eq 3 was applied to the case study database mentioned above. The following variables were considered: μ_max_ (1/h), lag phase (h), CDW (g/L), biomass yield (g biomass/g consumed substrate), and ethanol yield (g produced/g consumed). Robustness and performance values were calculated and plotted in R. Statistical difference among the different cellular functions and strains was determined with an unpaired, two-sided *t* test and *p*-values were adjusted with Holm–Bonferroni method.

Scripts with line-by-line explanation available on Github (https://github.com/cectri/Quantification-of-microbial-robustness).

## Supporting Information Available

Robustness of ethanol yield, eq 1 (Figure S1); Robustness of biomass yield, eq 2 (Figure S2); Mineral solution composition (Table S1); Vitamin solution composition (Table S2); List of chemicals (and their relative concentrations) added to the medium to mimic the composition of lignocellulosic hydrolysates (Table S3) (PDF)

## Notes

The developed scripts for robustness quantification are available at Github: https://github.com/cectri/Quantification-of-microbial-robustness.
