# Supplementary material for: Quantification of Microbial Robustness in Yeast
Source: ACS Synth Biol. 2022 Mar 11;11(4):1686–91. doi: 10.1021/acssynbio.1c00615 (PMC9016762; doi:10.1021/acssynbio.1c00615)
Supplement: Supplementary file 1 — sb1c00615_si_001.pdf [file sb1c00615_si_001.pdf]

Supporting Information

Quantification of microbial robustness in yeast

Cecilia Trivellin[1], Lisbeth Olsson[1], Peter Rugbjerg[1,2,*]

[1]Department of Biology and Biological Engineering, Division of Industrial Biotechnology, Chalmers University of Technology, Gothenburg, 412 96, Sweden

[2]Enduro Genetics ApS, Copenhagen, 2200, Denmark

*Correspondence: rugbjerg@chalmers.se

Figure S1

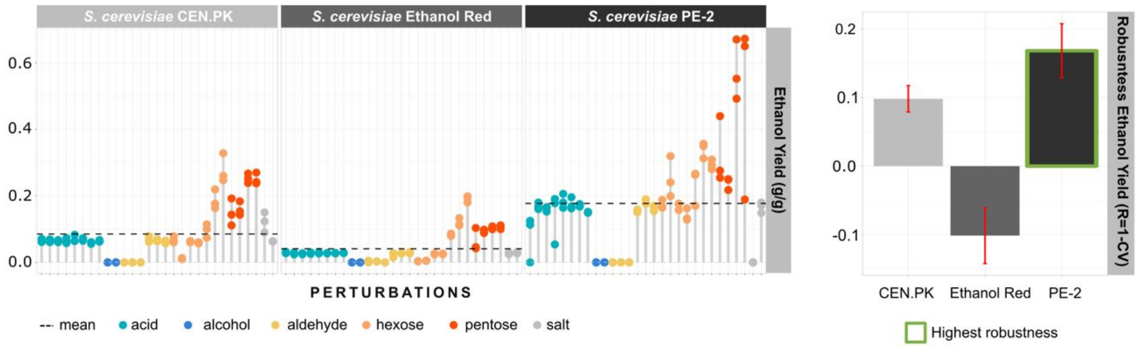

Figure S1: Robustness estimation with Eq1. The three panels to the left denote different S. cerevisiae strains grown in medium containing various components (grouped by color) that mimic lignocelluloses hydrolysates. Ethanol yield (g/g) measurements from the database created in the case study (see Material and methods) are reported. The plot to the right shows robustness of the ethanol yield presented in the left panels. Robustness (R) was calculated with Eq1 based on the coefficient of variation (CV) by applying the formula R = 1 - CV. Error bars correspond to the standard error of the mean.

Figure S2

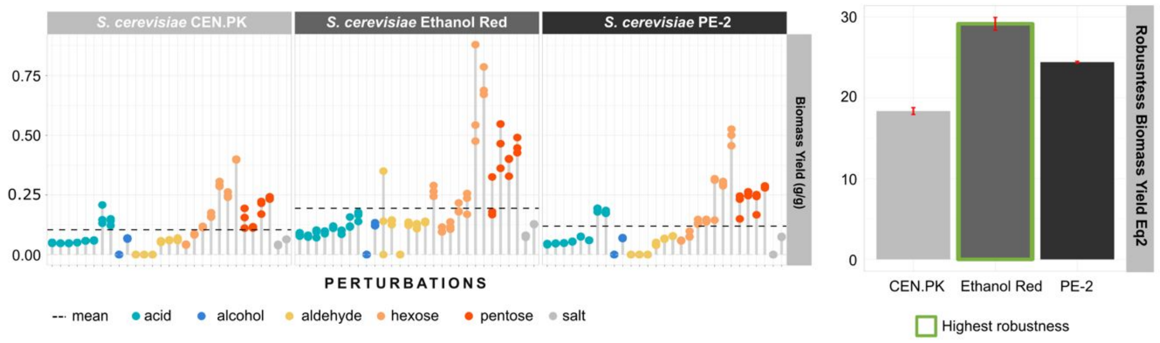

Figure S2: Robustness estimated with Eq2. The three panels to the left denote different S. cerevisiae strains grown in medium containing various components (grouped by color) that mimic lignocelluloses hydrolysates. Biomass yield (g/g) measurements from the database created in the case study (see Material and methods) are reported. The plot to the right shows robustness of the biomass yield presented in the left panels. Robustness was calculated with Eq2. Error bars correspond to the standard error of the mean.

Table S1: Mineral solution composition.

**Chemical**	**Amount (g/L)**
EDTA	0.015
$ZnSO_4 \cdot 7H_2O$	0.0045
$MnCl_2 \cdot 4H_2O$	0.0008
$CoCl_2 \cdot 6H_2O$	0.0003
$CuSO_4 \cdot 5H_2O$	0.0003
$Na_2MoO_4 \cdot 2H_2O$	0.0004
$CaCl_2 \cdot 2H_2O$	0.0045
$FeSO_4 \cdot 7H_2O$	0.003
H_3BO_3	0.001
KI	0.0001

Table S2: Vitamin solution composition.

**Vitamin**	**Amount (g/L)**
d-Biotin	0.00005
Calcium D(+) pantothenate	0.001
Nicotinic acid	0.001
Myo-inositol	0.025
Thiamine HCl	0.001
Pyridoxine HCl	0.001
Para-aminobenzoic acid	0.0002

Table S3: List of chemicals (and their relative concentrations) added to the medium to mimic the composition of lignocellulosic hydrolysates.

Chemical	Carbon source	Concentration (g/L)	Reference
D-(+)-Glucose		65, 30, 20	[1,2]
D-(+)-Xylose	D-(+)-Glucose (5 g/L)	36, 16	[1]
D-(+)-Galactose		4.5, 2	[2,3]
L-(+)-Arabinose	D-(+)-Glucose (5 g/L)	4, 2	[3,4]
D-(+)-Mannose		16, 10	[2,3]
Formic acid	D-(+)-Glucose (20 g/L)	3.5, 1	[1–3]
Acetic acid	D-(+)-Glucose (20 g/L)	6, 4.5	[1,3,4]
DL-Lactic acid	D-(+)-Glucose (20 g/L)	7, 2	[5,6]
Levulinic acid	D-(+)-Glucose (20 g/L)	5, 2.5	[2,3]
5-Hydroxymethylfurfural	D-(+)-Glucose (20 g/L)	6, 0.5	[1,2]
Furfural	D-(+)-Glucose (20 g/L)	3, 1	[1,3]
Vanillin	D-(+)-Glucose (20 g/L)	2, 0.5	[3]
Ethanol	D-(+)-Glucose (20 g/L)	90, 45	[7]
NaCl	D-(+)-Glucose (20 g/L)	80, 25	[8]

References

(1) van Dijk, M., Erdei, B., Galbe, M., Nygård, Y., and Olsson, L. (2019) Strain-dependent variance in short-term adaptation effects of two xylose-fermenting strains of Saccharomyces cerevisiae. *Bioresour. Technol. 292*, 121922.

(2) Palmqvist, E., Galbe, M., and Hahn-Hägerdal, B. (1998) Evaluation of cell recycling in continuous fermentation of enzymatic hydrolysates of spruce with Saccharomyces cerevisiae and on-line monitoring of glucose and ethanol. *Appl. Microbiol. Biotechnol. 50*, 545–551.

(3) Koppram, R., Albers, E., and Olsson, L. (2012) Evolutionary engineering strategies to enhance tolerance of xylose utilizing recombinant yeast to inhibitors derived from spruce biomass. *Biotechnol. Biofuels 5*, 32.

(4) Cavka, A., and Jönsson, L. J. (2013) Detoxification of lignocellulosic hydrolysates using sodium borohydride. *Bioresour. Technol. 136*, 368–376.

(5) Favaro, L., Basaglia, M., Trento, A., Van Rensburg, E., García-Aparicio, M., Van Zyl, W. H., and Casella, S. (2013) Exploring grape marc as trove for new thermotolerant and inhibitor-tolerant Saccharomyces cerevisiae strains for second-generation bioethanol production. *Biotechnol. Biofuels 6*, 168.

(6) Kim, D. (2018) Physico-chemical conversion of lignocellulose: Inhibitor effects and detoxification strategies: A mini review. *Molecules*. MDPI AG.

(7) Olsson, L., and Hahn-Hägerdal, B. (1996) Fermentation of lignocellulosic hydrolysates for ethanol production. *Enzyme Microb. Technol. 18*, 312–331.

(8) Hohmann, S. (2002) Osmotic Stress Signaling and Osmoadaptation in Yeasts. *Microbiol. Mol. Biol. Rev. 66*, 300–372.
